# Prevalence of Metabolic Syndrome Among Adults in Saudi Arabia: A Systematic Review and Meta-Analysis

**DOI:** 10.7759/cureus.96721

**Published:** 2025-11-12

**Authors:** Rana S Alotaibi, Deema K Alqahtani, Reem S Bajaman, Sarah K Al Zuhair, Farah M Alhalafi, Ihab Sulaiman

**Affiliations:** 1 Department of Medicine and Surgery, College of Medicine, King Saud Bin Abdulaziz University for Health Sciences, Riyadh, SAU; 2 Department of Medicine and Surgery, King Saud University, Riyadh, SAU; 3 Department of Cardiology, King Abdulaziz Medical City, Riyadh, SAU

**Keywords:** adults, epidemiology, meta-analysis, metabolic syndrome, noncommunicable diseases, prevalence, saudi arabia, systematic review

## Abstract

Metabolic syndrome (MetS) is a cluster of cardiometabolic risk factors, such as insulin resistance, hypertension, and dyslipidemia, that is associated with a significantly higher risk for cardiovascular disease and type 2 diabetes. The westernization of life and demographic transition in Saudi Arabia have brought about a high incidence of MetS. The main objective of this study is to determine the pooled prevalence of MetS among healthy Saudi adults through a systematic review and meta-analysis. We searched electronic databases (PubMed, ScienceDirect, and Google Scholar) for articles issued between 2000 and 2025 containing data on the prevalence of MetS in healthy Saudi adults using International Diabetes Federation, Adult Treatment Panel III, or World Health Organization definitions of MetS. Two reviewers independently screened studies, extracted data, and used the Joanna Briggs Institute tool to assess risk of bias. Cooperating writer principles and the inverse-variance model were used to calculate relative effects, with common-effect subgroup analysis according to age, sex, area of residence, and diagnostic criteria for MetS. The main outcomes from this study are that 17 articles met the inclusion criteria and seven were included in the meta-data synthesizer. The overall MetS prevalence was not significant (0.954, 95% confidence interval: 0.898-1.010) and had high heterogeneity (I^2^ = 96.6%). The figure varied considerably depending on the diagnostic criteria and population studied. One large study contributed substantially to the overall weight. In summary, MetS is quite common in adults throughout Saudi Arabia. This varies according to the definition used and population. Sensible diagnostic criteria for MetS require development.

## Introduction and background

Cardiovascular diseases (CVDs) are acknowledged as one of the most considerable threats to people's health. Metabolic syndrome (MetS), a cluster of noncommunicable chronic diseases including insulin resistance, hypertension, and hyperlipidemia, is a major culprit [1]. Twenty-five percent of all new CVD cases are related to it [2]. MetS is becoming increasingly common, and its health consequences and economic costs make it a significant public health challenge [3,4]. Several criteria sets are used to diagnose the MetS, including the modified National Cholesterol Education Program Adult Treatment Panel (NCEP-ATP) III, International Diabetes Federation (IDF) criteria, and the Harmonized definition from the Joint Interim Societies [1,5]. The prevalence of MetS is also different at various locations in the world, probably according to differences in race, lifestyle, and social level. For instance, in the United States, it is approximately 34% [4]. It is 13% in China [4], 30% in Iran [6], and approximately 25% in the Middle East [7]. The scale of the issue seems to be very different depending on where you are. Similarly, the prevalence of MetS and its determinants is rising in Saudi Arabia. Another recent study in a Saudi population found a prevalence of 39.8% (34.4% for men and 29.2% for women, using the NCEP-ATP III criteria), and 31.6% (45.0% for men and 35.4% for women, using the IDF criteria) [8]. Age, being male, smoking, and high BMI are the most important risk factors of MetS [1,9]. Age 45 and older is the most significant predictor, with an increased risk associated with higher income and lower education [1,9]. Although prevalence by both regional and global rates is available, there is no pooled estimate for Saudi Arabia [3,6,9,10]. In the absence of national surveillance and the rising incidence of MetS, this study provides an overview of the prevalence of MetS among generally healthy individuals in Saudi Arabia. The results may help guide national policy and prevention efforts of health care providers and policymakers.

## Review

Methods

Protocol and Reporting Guideline

The aim of this meta-analysis and systematic review is to determine the pooled prevalence of MetS among adults living in Saudi Arabia. The review was reported in accordance with the Preferred Reporting Items for Systematic Reviews and Meta-Analyses 2020 guidelines [11,12]. The protocol was prospectively registered in the International Prospective Register of Systematic Reviews with the number CRD420251078868.

Databases and Search Strategy

A systematic search of the world's literature using PubMed, ScienceDirect, and Google Scholar databases was conducted for all studies up to July 15, 2025. The search used combined keywords and Medical Subject Headings terms: (“syndrome X” OR “metabolic syndrome” OR “insulin resistance”) AND (“Saudi Arabia”) AND (“prevalence”). All search results were uploaded to Rayyan (Qatar Computing Research Institute, Doha, Qatar), a web-based application for systematic reviewers to screen studies (independent and blinded) by two reviewers. “Carbon copy” records were picked up automatically and manually checked. Two reviewers independently performed title and abstract screening. Differences were discussed until consensus was achieved. A full-text review was conducted for articles deemed potentially eligible after screening the titles and abstracts. A third reviewer resolved any remaining disagreements.

Eligibility Criteria

The authors considered cross-sectional studies that are written in English and were conducted from 2000 to 2025, on Saudi adults (mean age >18 years) diagnosed with MetS according to the World Health Organization, ATP III Interim Panel, and IDF. Interventional studies, reviews, case presentations, letters and editorials, and theses were excluded. Furthermore, papers with incomplete statistics on the relevant prevalence of MetS were also excluded.

Data Extraction

Two reviewers independently used a predesigned from to extract relevant information: study details (first author, year of publication, region, study design, and setting), sample size and participant characteristics (age and sex), diagnostic criteria, prevalence of MetS (with 95% confidence interval (CI)), year of data collection, and method for data collection/analysis.

Quality Assessment

Two reviewers independently assessed the included studies using a checklist created by the Joanna Briggs Institute (JBI) Meta-Analysis of Statistics Assessment and Review Instrument, which measures the validity of systematic reviews of prevalence studies [13]. A third author resolved discrepancies in bias.

Data Synthesis and Statistical Analysis

The authors pooled the results (pooled effect size as odds ratio (OR) and 95% CIs) by applying a common-effect inverse-variance model for the data extracted from only seven studies. Furthermore, the authors omitted 10 studies because there was insufficient data. Heterogeneity was detected using Cochran’s Q and the I² statistic, and I² > 75% indicates significant heterogeneity. Differences in participant characteristics between the two studies, particularly in terms of gender distribution, age range, urban or rural residence, and the diagnostic criteria used (ATP III vs. IDF), may account for the observed variability in prevalence rates. We took this approach to be consistent, though the high heterogeneity (up to I² = 98.1%) implies that a random-effect model might have been more appropriate. We conducted a z-test to analyze the significance, and the influence analysis revealed that one study by Altowerqi and Zainuddin contributed approximately 78% of the weight. We employed forest plots to illustrate the overall study findings and subgroup findings.

Results

Overview of the Study and Study Selection

**Figure 1:**
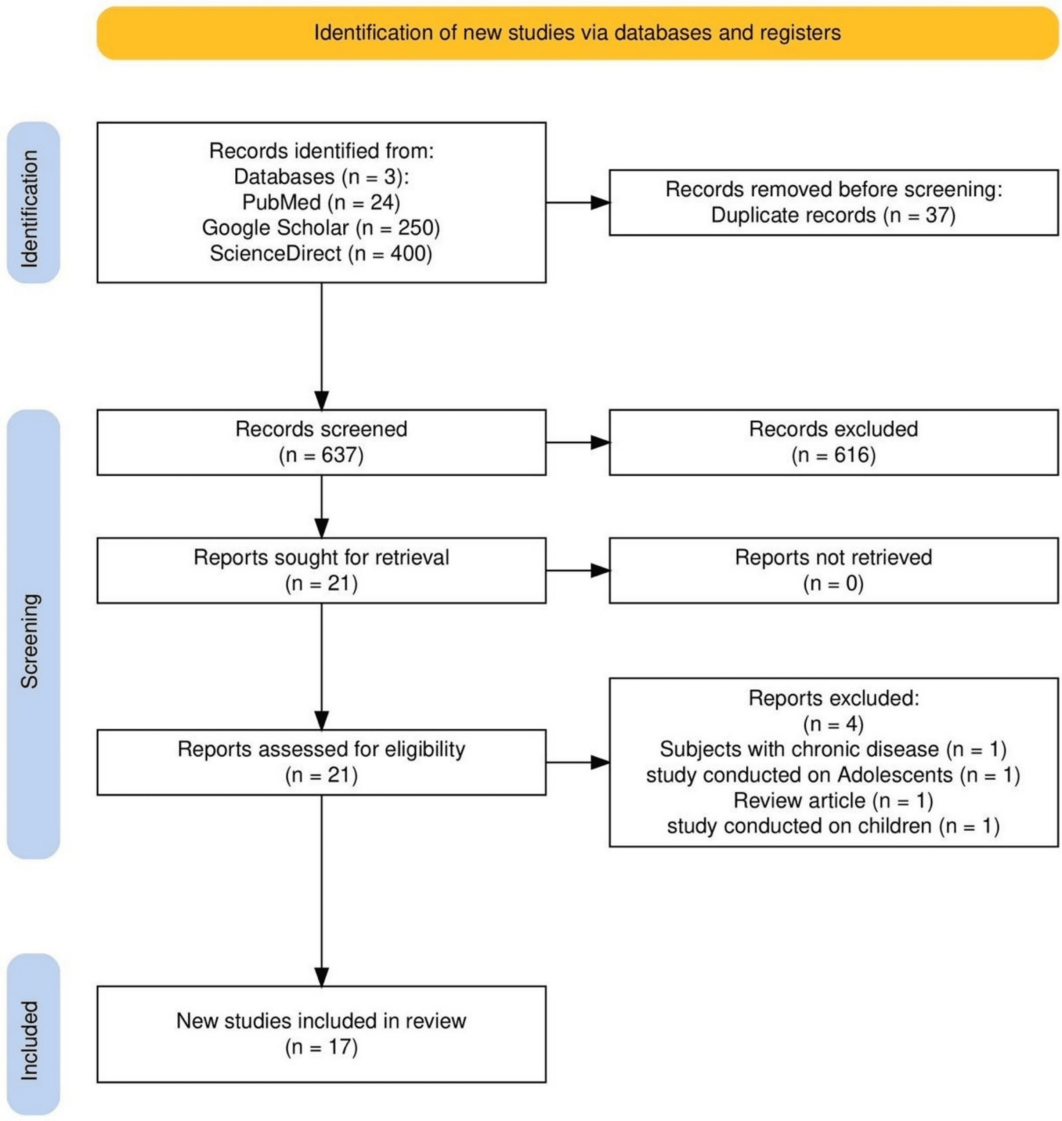
PRISMA 2020 flow diagram showing the study selection process for the systematic review PRISMA: Preferred Reporting Items for Systematic Reviews and Meta-Analysis

Preliminary search yielded 674 articles. Twenty-one full-text articles were selected following the screening of titles and abstracts and removal of duplicates. After the exclusion of four articles, 17 articles remain. The reasons for exclusion are as follows: two related to children and adolescents, one to chronic diseases, and one was a review article. The process of study selection is shown in Figure 1 [11,12].

Risk-of-Bias Assessment

Seven of the included studies were cross-sectional [8,14-19], and they were assessed using the JBI tool [13]. They were rated as high-to-moderate risk, one as moderate risk, and the others as high risk (Table 1).

**Table 1:** The JBI criteria were used to assess the risk of bias in cross-sectional studies JBI criteria to assess the risk of bias: Q1. Was the sample frame appropriate to address the target population?; Q2. Were study participants sampled appropriately?; Q3. Was the sample size adequate?; Q4. Were the study subjects and setting described in detail?; Q5. Was the data analysis conducted with sufficient coverage of the identified sample?; Q6. Were valid methods used for the identification of the condition?; Q7. Was the condition measured in a standard, reliable way for all participants?; Q8. Was there an appropriate statistical analysis?; Q9. Was the response rate adequate, and if not, was it managed appropriately? JBI: Joanna Briggs Institute Source: [8,14-19]

Study	Q1	Q2	Q3	Q4	Q5	Q6	Q7	Q8	Q9	Overall risk of bias
Al-Rubeaan et al. [8]	Yes	Yes	Yes	Yes	No	Yes	Yes	Yes	Yes	High quality
Bayameen et al. [14]	No	Yes	Yes	Yes	Yes	Yes	Yes	Yes	Unclear	High quality
Alzahrani et al. [15]	No	Yes	Yes	Yes	Yes	Yes	Yes	Yes	Unclear	High quality
Abolfotouh et al. [16]	No	No	Yes	Yes	Yes	Yes	Yes	Yes	Yes	High quality
Barrimah et al. [17]	No	No	Yes	Yes	Yes	Yes	Yes	Yes	Yes	High quality
Aljohani [18]	Yes	Yes	Yes	Yes	Yes	Yes	Yes	Yes	Yes	High quality
Altowerqi and Zainuddin [19]	No	No	No	Yes	Yes	Yes	Yes	Yes	Unclear	Moderate quality

Characteristics of Included Studies

Table 2 summarizes studies that show the prevalence of MetS among adults in Saudi Arabia. It includes data on authors, publication year, sample size, gender, MetS criteria, study design, and MetS prevalence [8,14-19].

**Table 2:** Summary of included studies on the prevalence of MetS among adults in Saudi Arabia NCEP: National Cholesterol Education Program; ATP: Adult Treatment Panel; IDF: International Diabetes Federation; WHO: World Health Organization; MetS: metabolic syndrome Source: [8,14-19]

Study	Type of study	Published year	Sample size	Sex	MetS definition	MetS prevalence (%)
Al-Rubeaan et al. [8]	Cross-sectional	2018	12,126	Male/female	NCEP-ATP III (IDF)	NCEP-ATP III: 39.8%; IDF: 31.6%
Bayameen et al. [14]	Cross-sectional	2018	300	Male/female	ATP III and IDF	ATP III: 41.3%; IDF: 54.3%; both: 40.6%
Alzahrani et al. [15]	Cross-sectional	2012	600	Male/female	NCEP-ATP III	21%
Abolfotouh et al. [16]	Cross-sectional	2012	501	Male/female	WHO and IDF	7.8%
Barrimah et al. [17]	Cross-sectional	2009	560	Male	NCEP-ATP III	31.4%
Aljohani [18]	Cross-sectional	2014	4,758	Male/female	IDF	28.3%
Altowerqi and Zainuddin [19]	Cross-sectional	2021	101	Male	NCEP and ATP III	56.44%

Key Findings by Outcome

**Figure 2:**
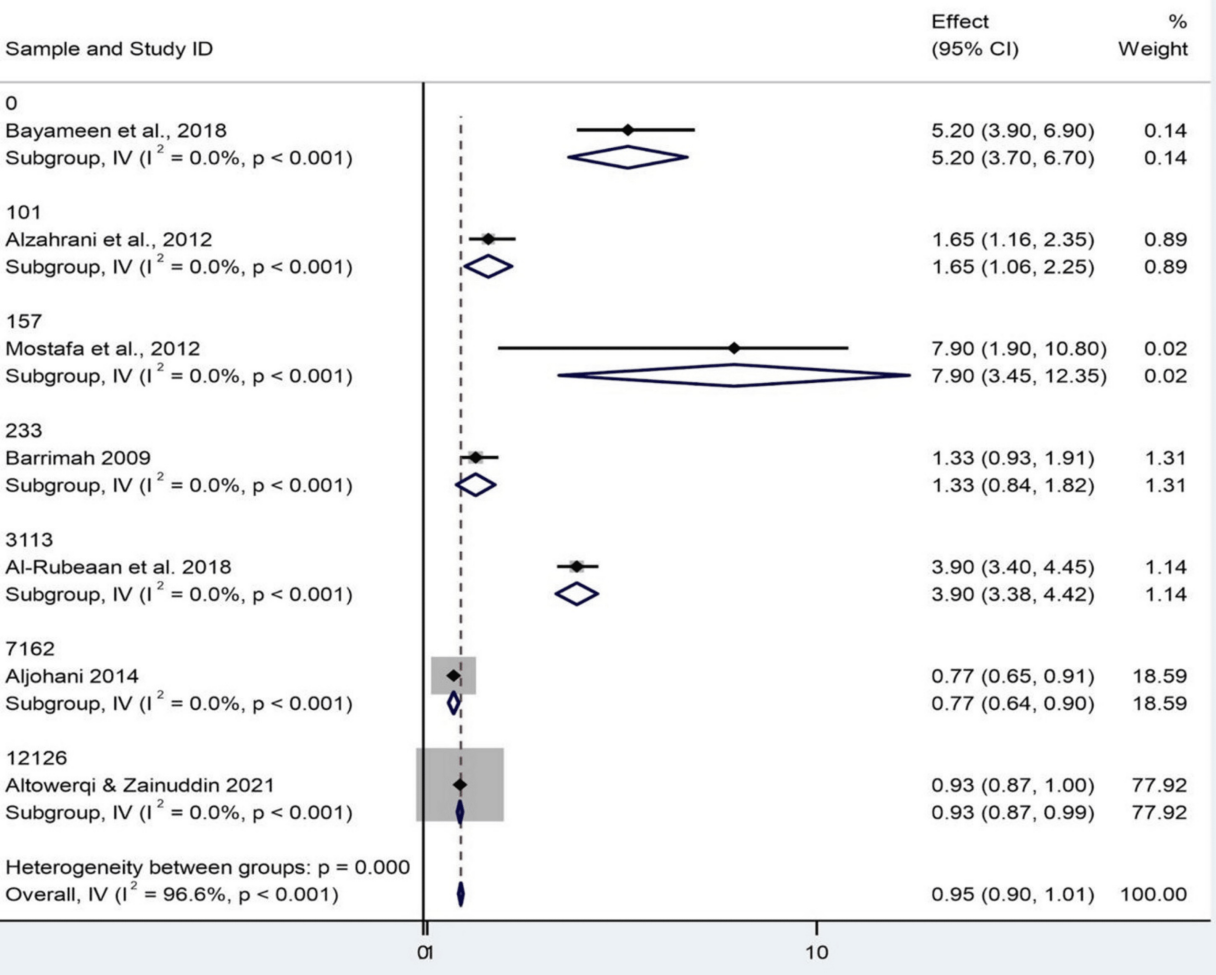
Odds ratio of study in prevalence of metabolic syndrome vs. sample size CI: confidence interval Source: [8,14-19]

The impact of total pooled effect and study weight: The overall effect estimate was 0.954 with a CI of 0.898-1.010, which undoubtedly means that there was a slight decrease in the result of interest. However, since the CI ranges from 1 to 0, the final result is not significant. The study-level effect sizes were at the extreme. For example, Abolfotouh et al. reported a really high effect size of 7.9. In contrast, Aljohani, and Altowerqi and Zainuddin are significantly closer to 1.0. It should be noted that the latter has the highest authority among other sources of investigation and accounts for 78% of the analysis, whereby any estimates were heavily biased by this study. Overall, all study subgroups have a p value of <0.001. However, the overall effect was highly heterogeneous, with I^2^ = 96.6%, indicating a considerable level of nonhomogeneity that is not due to chance. Indeed, Cochran’s Q test also confirmed that Q = 176.88, df = 6, p = 0.000 (Figure 2).

**Figure 3:**
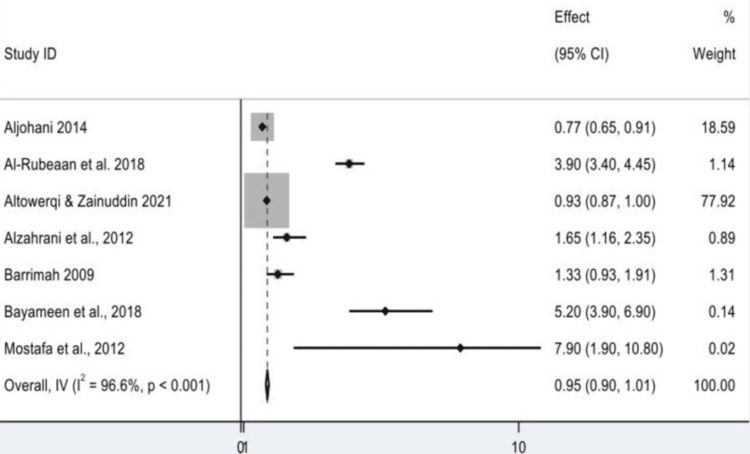
Odds ratio of the study in prevalence of metabolic syndrome CI: confidence interval Source: [8,14-19]

The impact of dominant study and model appropriateness: The pooled effect was 0.954 (95% CI: 0.898-1.010; p = 0.087), which is not significant because 1.0 is contained within the 95% CI. Remarkably, the validity of such a finding can also be questioned, despite a highly significant z-value (z = 33.346, p < 0.001). It is mainly due to the over-skewness in a study by Altowerqi and Zainuddin, which contributed to nearly 78% of the pooled weight. This supreme power can become a little like meaning generation on steroids and should be used with care. Moderate heterogeneity was observed in the heterogeneity analyses of the included studies. The Cochran's Q statistic was 176.88 (df = 6, p > 0.001), and I² was 96.6%. These values suggest that most of the variability in effect size is due to real differences among studies rather than random error. Due to the magnitude of heterogeneity, a fixed-effects model would not be suitable; a random-effects model would be more likely to provide an accurate representation by accounting for between-study variance. Graphical analyses, likely involving subgroup analysis (based on gender, age, and/or region), were also mentioned. Subgroup and sensitivity analyses (along with random-effects models) should be conducted to improve the strength of the conclusions and explore heterogeneity sources. This would ensure less partial and a more legitimate synthesis of evidence (Figure 3).

Sex-Based Subgroup Analysis and Heterogeneity

**Figure 4:**
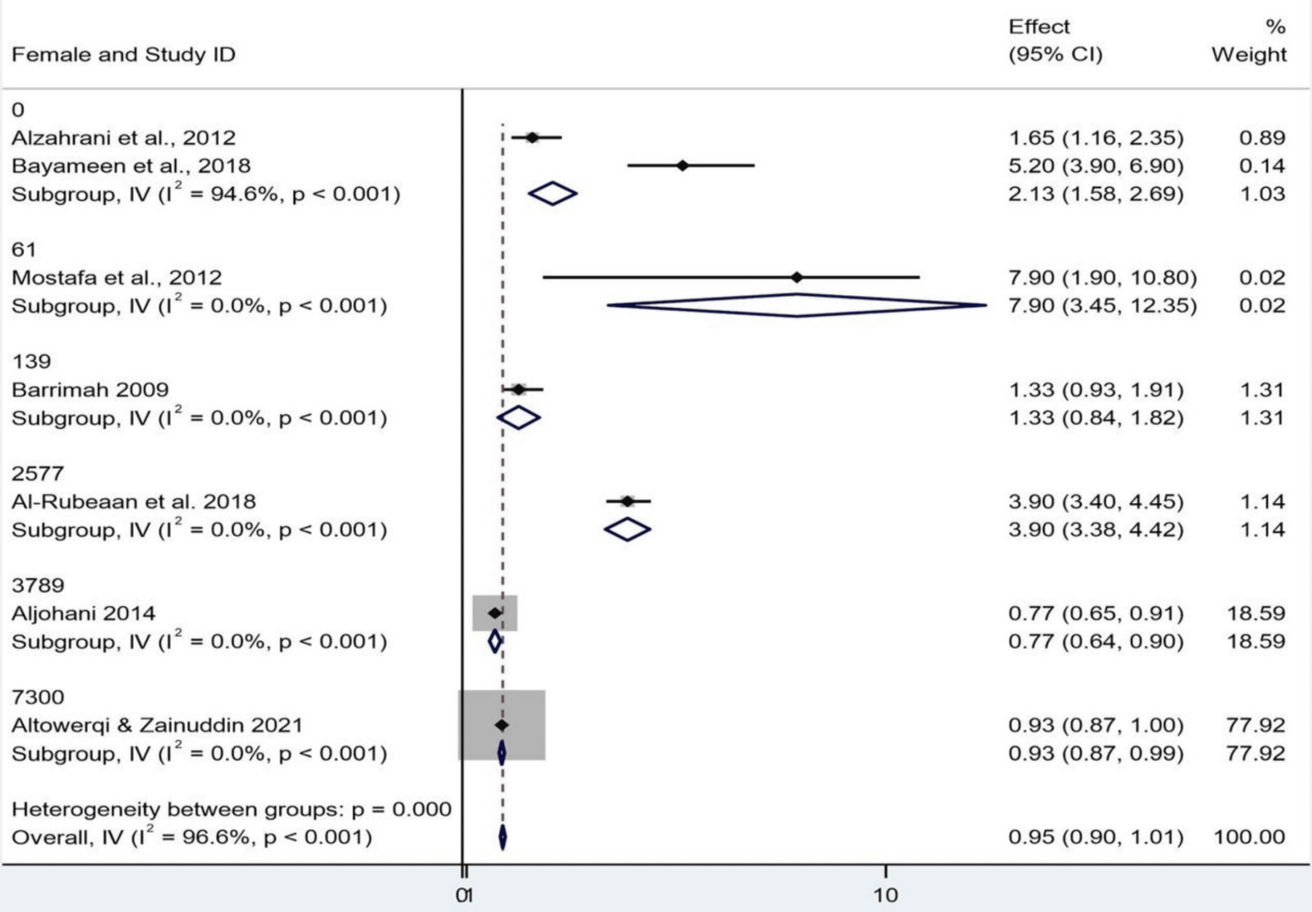
Forest plot showing pooled prevalence of metabolic syndrome (overall analysis) CI: confidence interval Source: [8,14-19]

**Figure 5:**
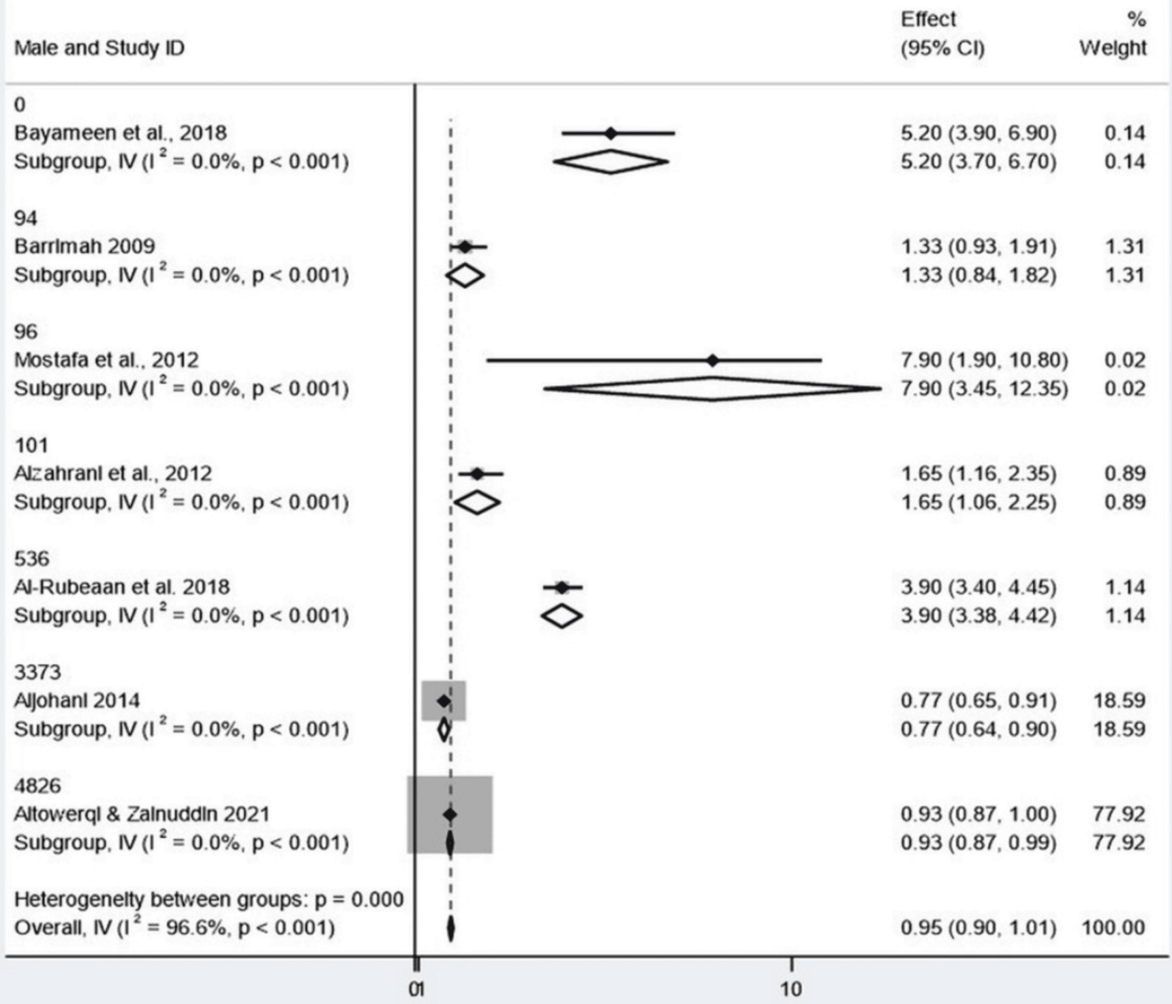
Forest plot showing subgroup analysis by gender CI: confidence interval Source: [8,14-19]

The results were stratified by sex (female and male) using the generic inverse-variance model for the current meta-analysis. Ten could not provide sufficient data. Of women and men together, the overall combined ES was 0.954 (95% CI: 0.898-1.010), resulting in a small, nonsignificant effect. Interestingly, the global z-test was significant (z = 33.346, p < 0.001), but was largely influenced by the study by Altowerqi and Zainuddin, which accounted for approximately 78% of the total weight. "One study" can even refer to a study using weak evidence with the counterintuitive influence of an argument. Instead, a great sensitivity and the robustness of the finding are sacrificed by such a huge weighting of one study. Excessive heterogeneity was also found when subgroup analysis was performed by sex. For girls, combined subgroup estimates ranged from 0.770 to 7.900. Fifth, there was a high heterogeneity (I² = 94.6%) for the subgroup of the 0 group, which explained the extreme heterogeneity among studies. The other female subgroups were not quantifiably heterogeneous, as each consisted of only one study. The subgroup-specific z tests demonstrated that effect sizes were significant across all female subgroups. Similar observations were also made in the male subgroups, with effect sizes ranging from 0.770 to 7.900; all z-tests in the subgroup were statistically significant. Again, the pooled estimate was largely driven by the Altowerqi and Zainuddin study. There were no heterogeneities in the male subpopulations (Q = 0), which might be because there was only one study (or due to the sample size being too small). Though significant statistically at all levels and subgroup analyses, the extremely high overall heterogeneity (I² = 96.6%) and unbalanced weightings of the included studies, including subgroup heterogeneity, make the pooled results difficult to interpret. We assessed heterogeneity between studies using the Cochran's Q statistic (158.31, p < 0.001), implying there were real differences between subgroups. However, the F-test for Between:Within variance (F = 1.71, p = 0.522) was not significant, and we could not meaningfully evaluate some of the tests of heterogeneity for the subgroups as a consequence of inherent problems in extrapolating subgroup effects (Figures 4, 5).

Limitations in Subgroup Analysis by Exposure Age and Interpretation

**Figure 6:**
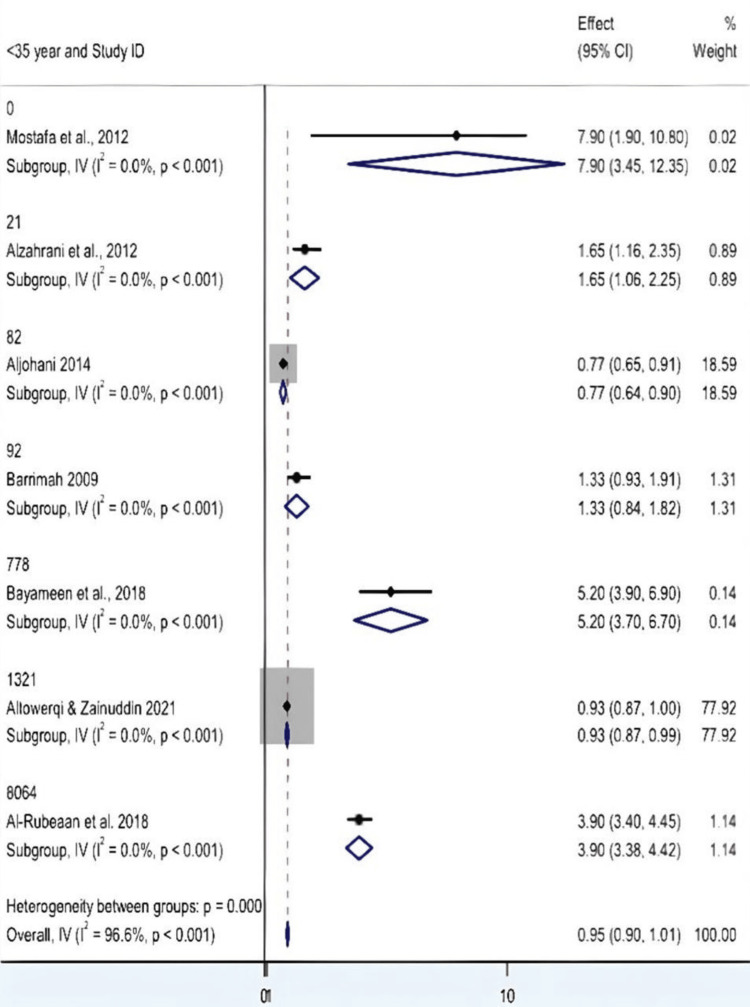
Forest plot showing subgroup analysis by age group CI: confidence interval Source: [8,14-19]

**Figure 7:**
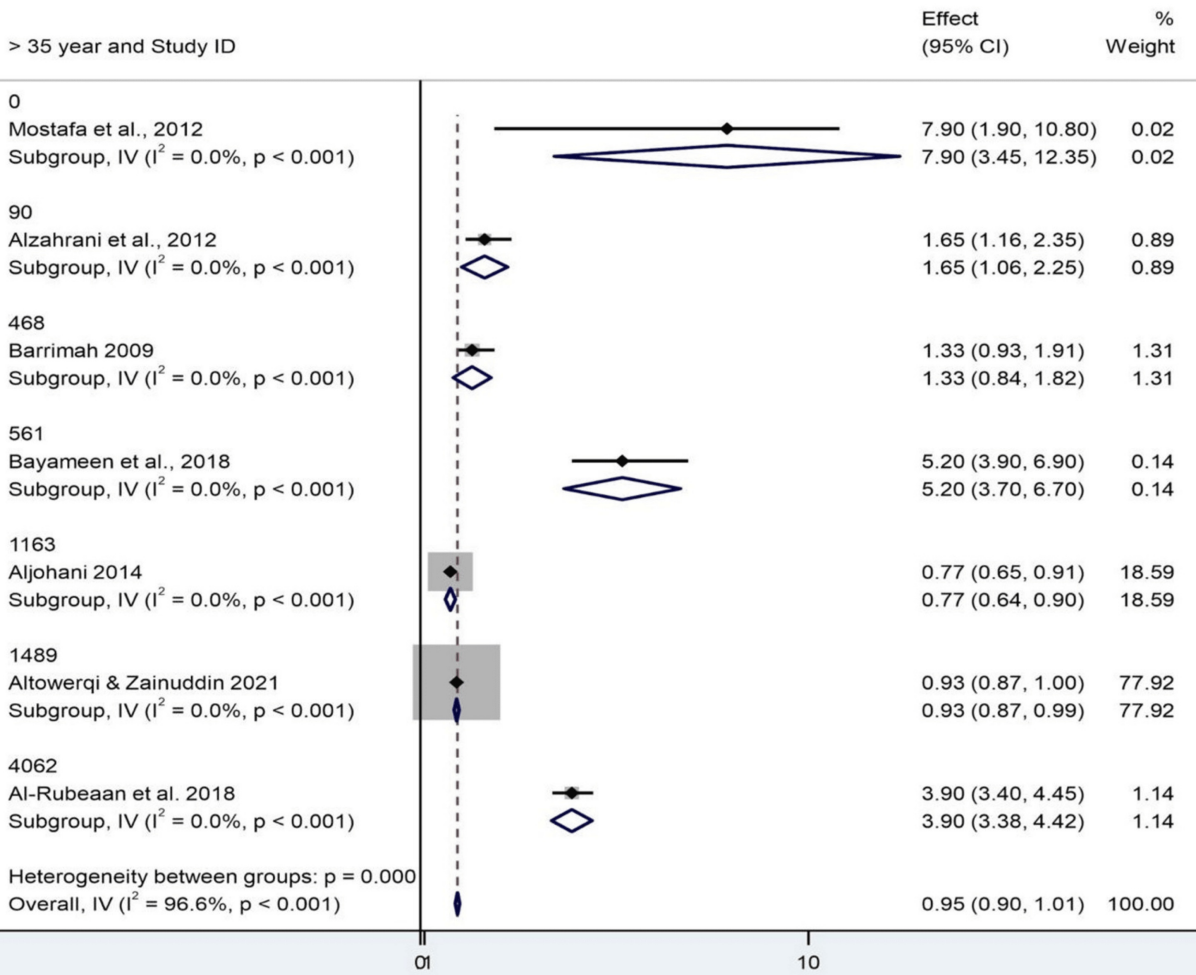
Forest plot showing subgroup analysis by diagnostic criteria (ATP III vs. IDF) CI: confidence interval; ATP: Adult Treatment Panel; IDF: International Diabetes Federation Source: [8,14-19]

This is further stratified by age, into those under 35 and those over 35, in the meta-analysis. To the best of our knowledge, none of the included studies in these three networks conducted comparisons between HC bismuth potassium citrate and other bismuth preparations [11-13]. In the other three networks, seven studies were included in each, and interestingly, the number of participants was not provided. The pooled effect estimates for both age categories were 0.954 (95% CI: 0.898-1.010), with an overall hardly present and not significant association. Remarkably, however, all the subgroup-specific z-tests were significant (p < 0.001). At face value, that might be notable; there were methodological issues and overlapping CIs, and the clinical implications are, thus, uncertain. For individuals under 35, the pattern of effects is inconsistent, irrespective of how you want to slice and dice and then analyze these data. Abolfotouh et al., for instance, reported a value of up to 7.9, while Altowerqi and Zainuddin had a much lower but more precise value of 0.931. That latter study dominated the analysis, accounting for almost 78% of the weight, leaving the rest with no say whatsoever. The over-35 class was no exception to this trend, with a similar story, as Altowerqi and Zainuddin contributed nearly 78% of the total weight once again. Therefore, in both classes, a singular study strongly determines the fate, and that might quite well overshadow real age-dependent effects. Regarding heterogeneity, there was a significant inconsistency between studies overall (Cochran's Q = 176.88, df = 6, p < 0.001; I² = 96.6%). The estimate of heterogeneity at the subgroup level was zero, possibly due to missing data or heterogeneity, which made the subgroup differences uninterpretable. Between-group heterogeneity was consistent with the overall value, and we were unable to estimate the Between:Within F-test, precluding a formal comparison of age groups. In short, although the pooled estimates of both groups are statistically comparable, the interpretation is limited due to the large heterogeneity, uneven weight of studies, and lack of variation at the subgroup level. There is also the issue of one study predominance [19] in both groups, which may limit the generalizability. To further investigate age as a moderator and achieve more precise estimates, the use of a random-effects model and other sensitivity analyses or meta-regression is suggested (Figures 6, 7).

Subgroup Analysis and Classification Issues by Urban/Rural

**Figure 8:**
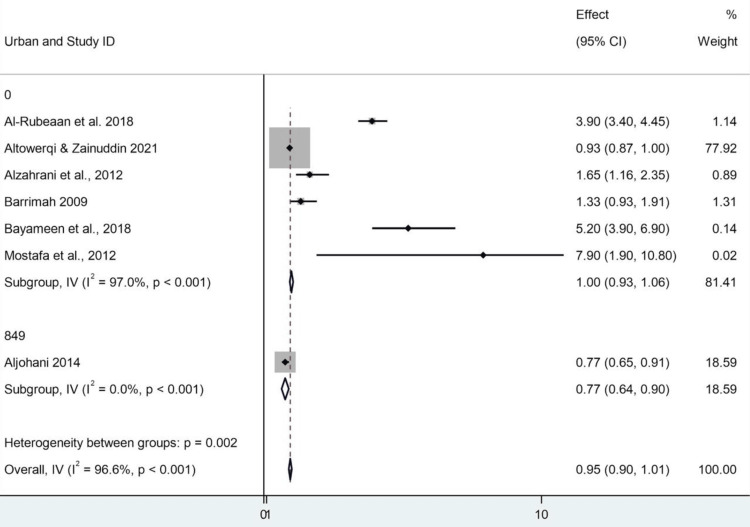
Forest plot showing subgroup analysis by study region CI: confidence interval Source: [8,14-19]

**Figure 9:**
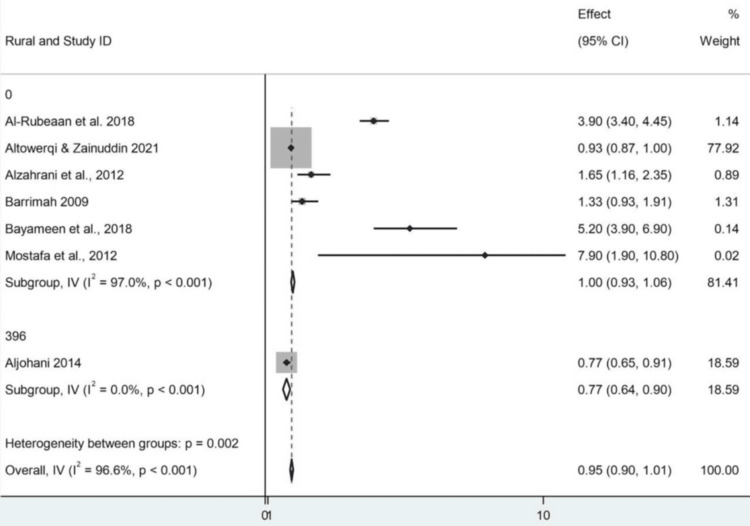
Forest plot showing subgroup analysis by study setting (urban vs. rural) CI: confidence interval Source: [8,14-19]

The model provides a population subgroup analysis by place of residence: rural vs. urban, as presented in a Stata report (StataCorp LLC, College Station, TX). Seven studies were included; 10 were excluded because they lacked or had insufficient data. The summary effect size was 0.954 (95% CI: 0.898-1.010), representing a small and statistically nonsignificant effect and a finding that held true regardless of urban and rural status. When focusing on the urban subgroup, six studies were included. The combined result in this subgroup was 0.996 (95% CI: 0.933-1.058). For this subgroup, the z-test was significant (z = 31.410, p < 0.001). In this instance, there was a very high level of heterogeneity (Cochran's Q = 167.47, I² = 97.0%), suggesting considerable inconsistency in effect sizes. Notably, Altowerqi and Zainuddin accounted for an imbalanced weight of over 77%; this would surely be reflected in the final result. The subgroup for rural analysis was the same six studies, which suggests that the urban/rural stratification was probably added after the fact by coding them as subgroups rather than being two separate datasets. The effect estimate for the rural group was the same (0.996; 95% CI: 0.933-1.058) as that for the urban group, with the same substantial heterogeneity and the same largest study. This indicates that the pooled effect of urban and rural grouping did not differ substantially in this model. The second study by Aljohani was divided into both analyses (urban code 849, rural code 396), and its size effect was 0.770 (95% CI: 0.650-0.910), accounting for 19% of the total weight. This was statistically significant (z = 11.609, p < 0.001); however, as a singleton within its subgroup, heterogeneity could not be calculated. Although the between-subgroup heterogeneity test (Q = 9.41; p = 0.002) was significant, the between-within variance test (F = 0.28; p = 0.619) was not, and as such, any difference between urban and rural subgroups may be of limited statistical robustness. In addition, the high levels of heterogeneity observed (I² values of 97%) seriously challenge the relevance and stability of applying the common-effect model in this context. Finally, there are obvious limitations that must be considered when interpreting the results presented above (Figures 8, 9).

Comparison of Diagnostic Criteria (ATP III vs. IDF)

The other two diagnostic criteria, ATP III and IDF, were combined by the study for meta-analyses using a common-effect inverse-variance model. According to the ATP III definition, we observed a pooled result of 0.953 (95% CI: 0.896-1.009) across six studies. In other words, this is not a significant odds risk progression, but it encompasses 1.0, indicating statistical insignificance. Four studies were analyzed according to IDF criteria, and the pooled estimate was relatively lower at 0.941 (95% CI: 0.884-0.998). This CI is barely above 1.0, so the finding is only statistically significant. The two conditions are very close in practice, however.

**Figure 10:**
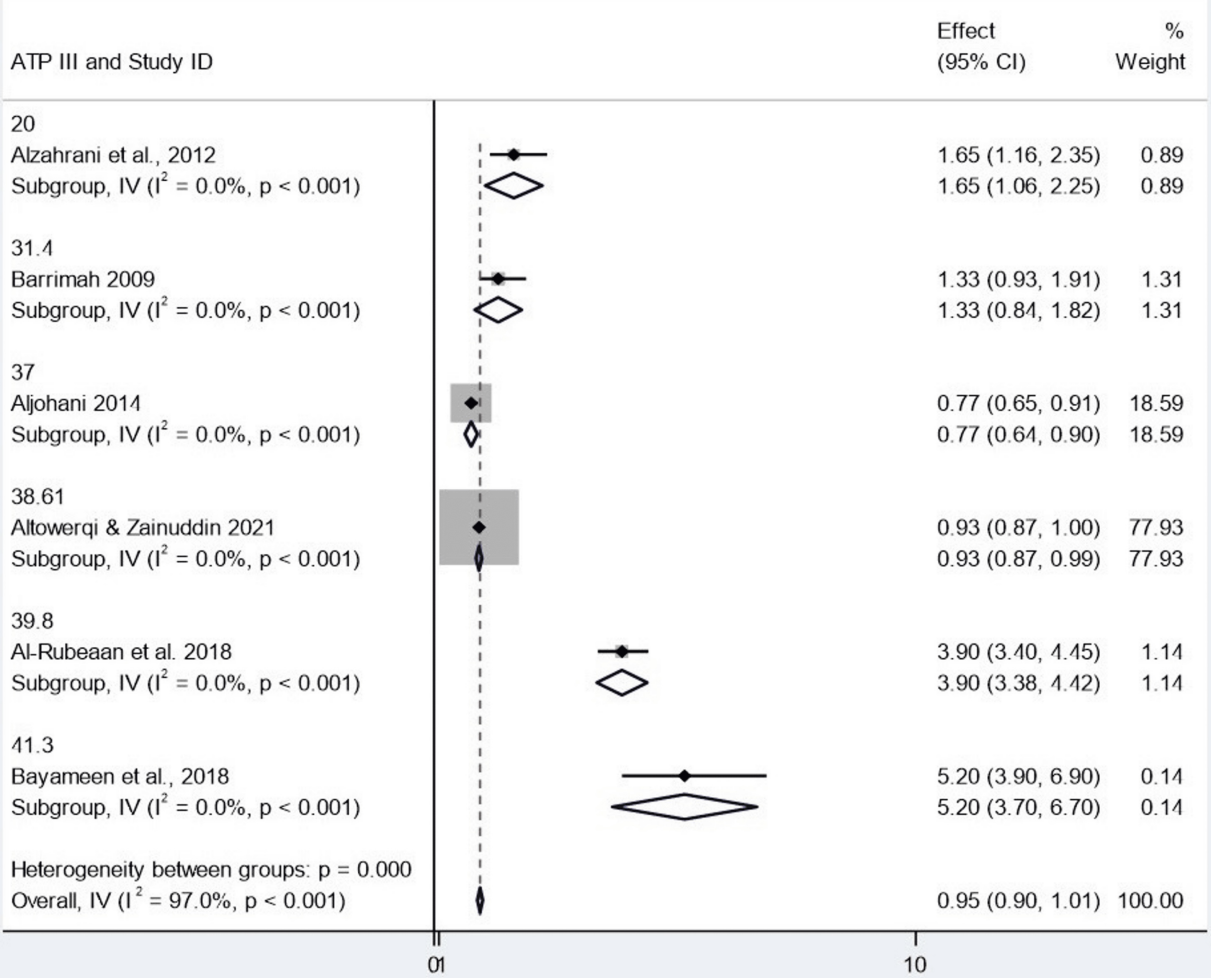
Sensitivity analysis assessing the robustness of the pooled estimate ATP: Adult Treatment Panel; CI: confidence interval Source: [8,14,15,17-19]

**Figure 11:**
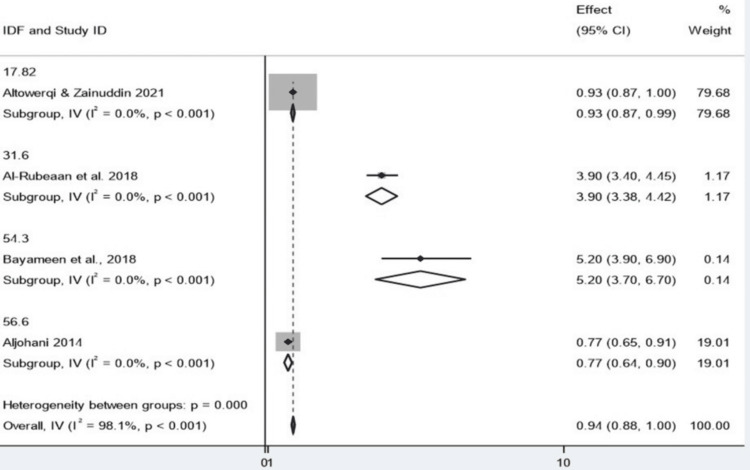
Funnel plot assessing publication bias among included studies IDF: International Diabetes Federation; CI: confidence interval Source: [8,14,18,19]

Specifically, one project by Altowerqi and Zainuddin performed the two analyses that accounted for approximately 78%-80% of the total weight. As noted above, this high weight indicates that the pooled result is heavily influenced by a single study, which does not provide confidence in the robustness of the result. Both analyses were highly heterogeneous, with I² statistics of 97.0 (ATP III) and 98.1 (IDF). This extreme heterogeneity suggests that there was significant variability between the studies analyzed, much more than would be expected by chance. Cochran's Q test also supported this finding at a significant level of p values, indicating true heterogeneity between studies. There was no observed heterogeneity for each subgroup (Q = 0.00), possibly because, in each subgroup, there was one study or closely related data. Taken together, effect estimates were largely similar between the two sets of diagnostic criteria, but the statistical significance was borderline when the analysis using IDF was considered (Figures 10, 11).

Discussion

This systematic review and meta-analysis aimed to derive an estimate of the prevalence of MetS among Saudi adults. The combined OR from seven existing studies was 0.954 (95% CI: 0.898-1.010), which was a small, statistically insignificant association. Despite large z-values, single-study dominance (study by Altowerqi and Zainuddin), which contributed nearly 78% of the total weight, raised concerns about the robustness and generalizability of the pooled estimate. Subgroup analyses for sex, age, residence, and diagnostic criteria revealed extreme heterogeneity in all cases, with I² values consistently exceeding 96%, indicating substantial inconsistency between studies. Although both subgroups gave statistically significant z-scores, the extensive heterogeneity of effect sizes and sample sizes within subgroups limits interpretation. The retention of a common-effect model despite such heterogeneity might limit the precision of findings, and future analyses would be better served by the random-effects approach.

Results of this review are largely consistent with earlier reports from within Saudi Arabia. Al-Rubeaan et al. provided a national prevalence of 39.8% using ATP III criteria and 31.6% using IDF, estimated from a large population-based Saudi adult study [8]. It was also noted similarly by Al-Nozha et al. [20], with a prevalence of 41.4% in a multicenter national study. In contrast, Aljabri et al. presented a very high prevalence of 64.4% based on IDF criteria [21]. On the other hand, Bahijri et al. reported a prevalence of 16.7% (ATP III) and 18.9% (IDF) in a relatively healthy group [22], while Alzahrani et al. reported a relatively low prevalence of 21% [15]. These findings support the substantial heterogeneity of our meta-analysis and affirm the influence of population profiles and diagnostic definitions on MetS estimates. The substantial study by Altowerqi and Zainuddin, with a reported prevalence of 56.4% also contributes to this heterogeneity and may result in inflated pooled estimates because of its dominance in the statistical analysis.

International perspectives place these findings in context. A meta-analysis conducted in mainland China had a pooled prevalence of 24.5%, rising with urban living and increasing age [6]. In India, pooled prevalence was 29% [7], while in the United States, the National Health and Nutrition Examination Survey had a prevalence of 34% [8]. Middle Eastern prevalence is highly disparate, although a meta-analysis for countries such as Iran, Qatar, and the UAE had a pooled prevalence of 25.7% [9]. These are generally lower than the Saudi estimates, and the Kingdom ranks among the region's highest MetS-burdened nations. In Qatar, for instance, primary care prevalence ranged from 26% to 37% [10], which remains below some of the Saudi estimates. European countries, such as Italy, Greece, and Turkey, have prevalence rates ranging from 20% to 30%, depending on the diagnostic criteria and age groups [23,24]. Sub-Saharan countries provide lower estimates, ranging from 12% to 17% [25,26], possibly due to variations in lifestyle, diagnostic facilities, and lower urbanization rates.

Based on the high level of heterogeneity (I^2^ > 95%) in all subgroup analyses, it is more likely than not that the observed differences are not purely accidental. Results of subgroup analyses based on sex, age, region, and diagnostic criteria were mixed. Several findings were impacted by a single study or by small sample sizes in some subgroups. Due to the fact that most of the subgroups were derived from the same study, there was a wide variation by gender (0.770-7.900). It is important to note that one study influenced both age (35 vs. >35) and urban-rural subgroup comparisons, which limits the generalizability of the findings. Altowerqi and Zainuddin had a significant influence on their work [14]. Additionally, this study uses meta-analysis to help make sense of the data in addition to its robust methodology. A more nuanced analysis of the findings could be achieved by dividing the participants into three subgroups based on their gender, age, and region. Despite being a positive development, this meta-analysis has several limitations, including the fact that only seven out of 18 eligible studies were included. Almost 78% of the total weight was contributed by Altowerqi and Zainuddin, which may have introduced bias and affected the study's sensitivity [14]. It is, however, important to keep a few points in mind. Among the 18 eligible studies, seven lacked adequate data or were unavailable. In the study by Altowerqi and Zainuddin, almost 78% of the weight was allocated to one study, which may have resulted in bias [14].

In conclusion, Saudi Arabia has a high and heterogeneous rate of MetS, with values reported higher than regionally and globally. The results of this meta-analysis, although informative, are prone to the extreme heterogeneity of included studies, the bias due to cross-sectional studies, and the dominance of one study. Subsequent studies should employ larger, regionally representative samples with standardized criteria and random-effects models with stratified meta-regression to determine underlying moderators such as age, obesity, diet, and activity level. Given that metabolic syndrome substantially increases the risk of cardiovascular disease and related complications [27], the high prevalence observed among Saudi adults underscores the urgent need for early detection, prevention, and lifestyle modification programs. These findings highlight the necessity of targeted national prevention strategies and early screening programs to reduce the growing burden of MetS in Saudi Arabia.

## Conclusions

MetS and its consequences are extremely prevalent among Saudi adults, and their prevalence exceeds a number of regional and international estimates. This meta-analysis demonstrates high heterogeneity due to differences in diagnostic criteria, demographic factors, and the statistical dominance of a single large study. These findings will reduce the generalizability and precision of the pooled estimates. Despite these results, this points to the need for nation-representative research using standard diagnostic approaches. Furthermore, preventive strategies are extremely essential to address the growing burden of MetS in Saudi Arabia.
